# A Grave National Deficiency.—I

**Published:** 1917-08-11

**Authors:** R. Fortescue Fox

**Affiliations:** Hon. Medical Director of the Red Cross Clinic in London for the Physical Treatment of Disabled Officers


### PHYSICAL ORTHOPÆDICS.

### A Grave National Deficiency.—I.

By R. FORTESCUE FOX, M.D. (Hon. Medical Director of the Red Cross Clinic in London for the Physical Treatment of Disabled Officers).

NOTHING is more familiar in these days than the sight of men disabled by wounds. Every hamlet and village in the land has its quota of them. They are gathered together in hundreds and thousands in every town. Many of these men are, alas! hurt beyond recovery; for them there is no prospect but a hopeless and helpless future. Happily, however, we have discovered that in the majority of cases the disablement is only of a temporary or partial nature, provided the right methods of treatment can be promptly employed. These methods include much that was unfamiliar in the days before the war. The elements of treatment must be adapted and combined, with experience and skill; and the long and tedious process of recovery, even in the most favourable of cases, will impose a serious tax upon the patience both of the wounded man and his medical adviser.

The restoration of wounded and wasted limbs wholly or in part to their functional activity is the aim and object of orthopædic treatment, in the new and larger sense of that term. The name "Orthopædics" as a branch of medical practice is no longer, as in former days, limited to the deformities of children. It has taken on a new and vast application to cover the disabilities and deformities induced by war.

The urgency of the question of the restoration of the wounded, first as a military policy and afterwards as a national policy, is now beginning to be realised. A great step forward—not the only step needed, but a great step—was taken last year when the Army Medical Department founded a new group of military orthopædic hospitals, primarily for those who are still soldiers, and for whom it is possible still to hope for a return to military duty. I venture to think it was a good policy that these special orthopædic hospitals and centres, including the great hospital at Whitchurch, near Cardiff, should have been placed, at all events so far as the surgical treatment is concerned, under the single direction and co-ordinating hand of an acknowledged expert. But we may well ask, Is it possible that even these great institutions, with their skilled staffs, can cope with the vast numbers of cases of disablement, both in soldiers and in ex-soldiers? More and still more will be needed. Nothing less than a gigantic effort is necessary to deal with disabled soldiers everywhere, and especially with the many thousands who are returning to their homes. Under such circumstances we must all agree that the governors of the King Edward VII.'s Hospital have been amply justified in attaching to the hospital a special department for this restorative treatment. The new department owes its initiation to the foresight and energy of Colonel Bruce Vaughan, and is in certain respects an improvement upon any previous installation.

I desire to call particular attention to the fact that those who have organised the new military orthopædic hospitals have recognised that orthopædic treatment is not entirely surgical. There is room for the physician also. These hospitals have been furnished, it is true, with every kind of surgical appliance, many of them of the greatest ingenuity, but they have also included a more or less complete installation for physical remedies. If now a wounded soldier is considered to be in need of remedial baths, massage, electrical applications, mechanical treatment or curative exercises, I understand that it is intended to refer him to one of these hospitals, or to a Command Depôt in which these physical methods are employed.

The orthopædic system in its new shape is therefore twofold. I cannot for one moment pretend to speak for the surgical side of restorative work, beyond recording my admiration for its achievement. But it appears to me that it is not too soon for the medical profession to take stock of the position now occupied by physical remedies in the treatment of disabled soldiers. I shall therefore ask you to consider with me the experience that we have already obtained, and especially to consider how in the near future these remedies can be best applied for the relief of the vest numbers of disabled men in our country.

And first let me ask, what are physical remedies? One of the natural laws which in our own time has thrown powerful light upon the constitution of the external world is known as the Law of the Correlation of the Physical Forces. It was just a statement in a simple form of the fact that all the great natural energies by which we are surrounded are related one to another and to a certain extent interchangeable one with another. They are not in this view of them to be considered as separate or isolated forces, but almost as different forms of one energy. That is a great and fundamental law. So, speaking in a broad sense, we may say that physical treatment aims at bringing the forces or energies by which we are surrounded in Nature into an effective and hopeful contact with the human body. The human body is fitted in every conceivable way to react or respond to the impressions that come to it from without. The sensitiveness of the body to heat and cold, and to all forms of radiant and electrical and mechanical energy, is sufficiently obvious in health. It is often much increased and modified in various ways in disease. As warm-blooded animals we are extremely sensitive to the impressions of heat and cold, whether applied to the whole body or locally. Physical treatment, then, founding itself upon the laws of reaction and response in the body to all these external impressions, seeks to discover how they can be made to relieve pain

☛ An address delivered at the opening of the orthopædic department of King Edward VII.'s Hospital, Cardiff, on July 6, 1917.

### PHYSICAL ORTHOPÆDICS (*continued*).

and distress, both physical and mental, to reduce fever, to fortify the body against infection, to improve the nutrition of wasted limbs, to restore or control the faulty circulation, and to remove effusions and deposits which impede the free movement of muscles and joints. Is it not obvious that those who aspire to direct physical remedies must traverse a wide field of knowledge, and that the closest observation is necessary in order to apportion with exactness the nature and degree of energy which is to be brought into contact with the body. There is no branch of medical treatment which makes a larger demand upon the philosophical observer and the practical physician.

Looking back to the Victorian age, we can now observe that the whole tendency of medical progress within recent years has been to bring physical remedies into more general use and esteem. Unfortunately, however, their employment in medicine although strongly rooted alike in reason and in popular confidence, still remains, to a large extent, empirical. Certain branches, it is true, have been carefully studied, such as the action of light and other forms of radiant energy upon the skin. X-rays and radium have created a new science of radiology. Electric currents and potentials have been very largely employed, and especially during the war, for wounded soldiers, but their action upon the body still offers a fruitful field for research. Remedial exercises and mechanical treatment are now recognised as possessed of enormous educational and medical value, but they cannot be said to be satisfactorily applied in practice, because their effect upon the body has been but little studied in our country. The same applies to the influence of climate in disease, which in a world-wide commonwealth of nations could readily be made a matter of scientific investigation. Lastly, it must be confessed that the action of baths and natural waters, including the operation of heat and cold and other physical forces in the bath, exerted upon the sensitive nerves that cover the surface of the body, is even more unfamiliar. The science of hydrology deals with the use of waters, internally and externally, in the prevention and cure of disease. They are admittedly powerful agents, but, even more than is the case with other branches of physical treatment, the current knowledge of the action of these powerful agents, from causes to which I shall presently refer, has been but slight and indefinite.

As I have already said, the practice of physical treatment covers a wide scope, and it is often necessary that one method should be combined or interchanged with another. Surface treatments by heat or cold are *combined* with massage, movements, or electricity. But the guiding principle and the secret of success in physical treatment is to produce a favourable reaction in the body, and only to employ such methods, alone or in combination, as will confirm and strengthen this favourable reaction.

In all the belligerent countries, and not least in the British Islands, physical remedies have been much employed during the present war. Let me refer first to the health resorts. Certain of the great Continental health resorts, which have sometimes been called "soldiers' spas," have long been celebrated for the good effects of their baths and waters in the healing of wounds and the removal of stiffness. Before the war I visited a number of these places. At Teplitz, in Bohemia, the courteous director told me that after Leipzig, the "Battle of the Nations," the wounded were carried to Teplitz for the natural thermal baths, and the cures then effected gave a lasting reputation to the waters.

Many examples of the value of natural baths for wounded and invalid soldiers, both in ancient and modern times, could be quoted, and in our own country magnificent work has been done during the present war with the thermal waters of Bath and Buxton, and by other forms of hydrological treatment at all the British spas (including those of Wales). There can be no doubt that when the case records come to be examined hereafter, it will be found that British health resorts have made a very substantial contribution to the restoration of the wounded.

But the experience of the last two years has also shown that remedial baths can be employed upon a large scale quite apart from the spas. Although the spas must always remain the chief centres of British hydrology, many forms of hydrological treatment can be given in the towns, in hospitals, and in camps. One of the most remarkable developments in the treatment of wounded soldiers during the last twelve months has been the extended use of remedial baths.

Permit me to describe briefly the baths that we have found most useful for military cases. There are three chief kinds of remedial baths, which differ widely in their action and uses. The first is a pure stimulant, and its type is the douche, in which the action of heat and water is supplemented by percussion. Alternating or contrasting temperatures are also employed in these stimulating baths, and powerfully add to the tonic effect. The second kind of bath is a pure sedative. This is exemplified by the still or flowing pool bath, often of long duration, in which the temperature of the water closely corresponds with that of the skin—that is, some five degrees below blood heat. The sedative pool, as it is now called, is largely used for disorders of the nerve centres and of the circulation. Lastly, there are the local baths for disabled limbs, usually at high temperatures. The whirlpool bath, which is the most interesting development in this direction, subjects the limb immersed in it to a kind of hydro-massage. The high temperature of the water, and its whirling movement, relieve the pain and muscular spasm, and promote nutrition and the absorption of inflammatory swellings by greatly increasing the circulation in the limb. We have found that many stiff and painful limbs can be moved or manipulated in the whirlpool bath without pain, and we have also found that the stiffness and swelling which so often follow fractures can be prevented by baths of this kind if they are used at an early stage.

(*Continued on page* 386.)

### PHYSICAL ORTHOPÆDICS (cont. from p. 380).

Nearly two years ago plans were drawn up for a model hydro-therapeutic installation, comprising these three principal types of baths, for certain of the Command Depôts. First in point of time the Command Depôt in Tipperary opened a sedative-pool bath in April of last year. This is a large pool, holding twelve to fourteen men, and has been extensively employed, especially for neurasthenia and shell-shock. A little later a complete installation of the three kinds of remedial baths were set up at the Northern Command Depôt at Heaton Park, Manchester. Rather more than a year has elapsed since these baths were taken into use. Good results have been reported from all three forms of treatment. The pool bath has been especially used in cases of disordered action of the heart. The whirlpool bath has also been much employed not only for wounded limbs, but for trench foot, and with excellent results. The limited accommodation at first provided in this department at Heaton Park has proved insufficient, and has now been doubled by the use of additional buildings.

